# Heart Cases in School Life

**Published:** 1912-10-05

**Authors:** 


### THE IDEAL SCHOOL CLINIC.

### Heart Cases in School Life.

Among the cases that will need the school doctor's most careful attention are those in which the children suffer from heart lesions. Such heart cases cannot be said to be common, but every school shows one or two of them. Usually what is found at inspection is that the child has a compensated degree of mitral regurgitation. Very rarely an aortic lesion is found. Much more common in school age is a presystolic apical bruit, which the inexperienced inspector, fresh from his hospital work, generally labels " mitral stenosis " and writes down as " heart disease." As a corollary to this diagnosis he probably informs the parent or teacher of the fact and tries to safeguard the child by restricting its play and school work. When he has listened to a few thousand hearts, however, he soon sees the necessity for modifying his views in regard to the apical presystolic murmur.

### The Presystolic Murmur.

Without entering into the discussion as to what exactly may be the meaning of these bruits in children, it may be stated that the discovery of such an abnormal sound cannot be accepted as proof of a definite cardiac lesion, necessitating restriction of school work or play. Re-examination has almost invariably failed to detect the murmur, and the children could, therefore, not be classed as suffering from heart disease. In such cases the parents and teachers naturally find the " clean sheet " offered by the re-inspector somewhat difficult to accept. Heart disease, they argue, cannot be present one day absent the next. For that reason it behoves the school doctor to be cautious in making a definite diagnosis of mitral stenosis; it is better to re-examine a child with such a murmur on a later occasion than to diagnose the condition at once merely on the strength of the presystolic murmur. It need scarcely be said that any child that shows such a murmur must be carefully examined—lying down, standing, and after exercise. The diagnosis of cardiac disease is not difficult if care is taken to eliminate possible factors which may influence the heart's action at the time of auscultation—nervousness and actual fright, in the case of young children, being particularly likely to lead to errors.

The case of the child who suffers from mitral regurgitation is on a different footing. Here no mistake is possible. The systolic murmur is generally pronounced, and in extreme cases there are the signs of failing compensation. These latter, of course, should at once induce the school doctor to exclude the child. In the classes one of the early signs of failing compensation is sickness and breathlessness after slight exertion, and a good rule is never to omit to examine the heart of a pupil who is stated to have been sick in class. Very often the sickness is due entirely to gastric irritation caused by over-indulgence, but in a few it has another cause which, unless the heart is carefully examined, is apt to be overlooked. Where obvious signs of failure are present—fainting, blue lips, flushed face, and back pressure symptoms—exclusion and rest must be insisted upon. Where, on the other hand, compensation is perfect it is unnecessary and undesirable to interfere more than is absolutely requisite with the child's school and home life.

The parents in such a case must be warned of the dangers that will undoubtedly follow failure of compensation. Considerable tact is necessary to impart this knowledge without frightening both parents and teachers, for the layman is apt to look upon " heart disease " as the most pernicious and pitiable condition. Where there is any real danger of prejudicing the child's health by school or home work there must be no hesitation in being frank with the parents and teachers. In some cases it may be necessary to recommend the child for a special school. This is particularly desirable when the daily attendance in class involves the climbing of several flights of stairs, an exertion which is by no means conducive to maintaining compensation. Games like football, water polo, and even cricket must be prohibited in all cases where there is marked breathlessness on slight exertion. Where compensation is well established it is possible that indulgence in such games, under the supervision of a careful master, is not likely to do much harm. Probably such exercise in these mild cases does not militate against the compensation more than does the average street play and yard exercise which every child will indulge in no matter how careful its parents may be.

### Should the Child be Told?

Whether or not the child should be warned is a matter that every school doctor will have to consider. There are some doctors who hold that it is inadvisable to tell the child of its condition. They point out that such knowledge is unnecessary and may make the child hypochondriacal. We do not think there is much in that argument. In the first place, the average intelligent boy or girl will be smart enough to follow what the doctor tells its parent, or if the information is given to the parent in private, to find out what is the gist of it afterwards. Secondly, when there is really any necessity for precautions the very fact that these are being taken will enable the child to know that there is something wrong. On the whole it therefore seems much better to take the child into one's confidence, to look for its co-operation, and to tell it frankly, in simple and common-sense language, what is the matter with its heart and in what way excessive indulgence in exercise or play may or will harm it. With the knowledge of its cardiac condition, the average intelligent boy or girl is in a far better position to guard against failure of compensation. In every heart case the school doctor should mark down on the card the nature of the condition found and the full particulars of the advice given to the parents or teacher. That precaution is in his own interest and in that of the school, and the note may be valuable evidence in case of some disaster to the child while at school.

